# TLTD: A Testing Framework for Learning-Based IoT Traffic Detection Systems

**DOI:** 10.3390/s18082630

**Published:** 2018-08-10

**Authors:** Xiaolei Liu, Xiaosong Zhang, Nadra Guizani, Jiazhong Lu, Qingxin Zhu, Xiaojiang Du

**Affiliations:** 1 School of Information and Software Engineering, University of Electronic Science and Technology of China, Chengdu 610054, China; liuxiaolei@uestc.edu.cn (X.L.); qxzhu@uestc.edu.cn (Q.Z.); 2 Cyberspace Security Research Center, University of Electronic Science and Technology of China, Chengdu 611731, China; 201411220203@std.uestc.edu.cn; 3 Department of Electrical and Computer Engineering, Purdue University, West Lafayette, IN 47907, USA; nguizani@purdue.edu; 4 Department of Computer and Information Sciences, Temple University, Philadelphia, PA 19122, USA; dux@temple.edu

**Keywords:** internet of things, traffic detection, adversarial samples, machine learning

## Abstract

With the popularization of IoT (Internet of Things) devices and the continuous development of machine learning algorithms, learning-based IoT malicious traffic detection technologies have gradually matured. However, learning-based IoT traffic detection models are usually very vulnerable to adversarial samples. There is a great need for an automated testing framework to help security analysts to detect errors in learning-based IoT traffic detection systems. At present, most methods for generating adversarial samples require training parameters of known models and are only applicable to image data. To address the challenge, we propose a testing framework for learning-based IoT traffic detection systems, TLTD. By introducing genetic algorithms and some technical improvements, TLTD can generate adversarial samples for IoT traffic detection systems and can perform a black-box test on the systems.

## 1. Introduction

In recent years, with the extensive applications of the Internet of Things (IoT) and the continuous development of machine learning algorithms, many researchers have proposed a number of IoT malicious traffic detection techniques based on machine learning algorithms [1,2,3,4,5,6]. However, most researchers only care about the performance of the detection model but ignore the vulnerability and robustness of the model. This makes the existing model vulnerable to “adversarial samples” [7]. It can make the model misjudgde and then enable the attacker to achieve the purpose of bypassing the model detection [8]. Such a model cannot be applied in practice. The adversarial sample is a special sample deliberately designed by the attacker. When it is input into the machine learning model, it can cause model classification errors. It is just like the visual illusion of the model. This type of adversarial sample designed for machine learning models has recently attracted the attention of many researchers [9,10,11]. For example, in the image classification system, by adding a slight perturbation to the original image, the change of the image classification result can be achieved with a high probability, and even the attacker can make the image be classified as an arbitrarily designated label [8]. Goodfellow et al. proposed a method based on fast gradient symbol algorithm to generate an adversarial sample [12]. Papernot et al. used the Jacobian matrix to determine which dimensions of information need to be modified when generating an adversarial sample [13]. In fact, the Jacobian matrix-based algorithm is also a gradient algorithm. Grosse et al. used a gradient-based algorithm to generate an adversarial sample of Android malware [14]. They assumed that the attacker can know the parameters of the malware detection model. For different neural networks, the model’s misclassification rate was shown to be 40% to 84% after processing the adversarial samples.

At present, most adversarial sample generation methods are designed for image classification models [15,16,17]. However, almost all machine learning models can be attacked by the adversarial samples. This means that there are also security risks in other areas of machine learning algorithm applications [18,19]. On the other hand, most of the current methods use gradient information to transform the original data into adversarial samples. If an attacker only knows what features the model uses, and he knows nothing about the parameters of the model, he cannot produce an effective adversarial sample [15,20].

This paper proposes a testing framework for learning-based IoT traffic detection system (TLTD). The main challenge of TLTD is the method of generating adversarial samples for the IoT traffic detection model based on a genetic algorithm. Without having to know the model parameters, TLTD uses the original data as the input of the algorithm, and produces the counter sample of the specific tag, and the only information used is the classification probability of the tag’s output by the model.

We hope TLTD can be a benchmark learning-based IoT traffic detection model. Our contribution is mainly reflected as follows:

1. We migrate the application scenarios of adversarial samples from the image recognition domain to the IoT malicious traffic detection field. This migration cannot be achieved simply by replacing the model’s training data from pictures to traffic. We need to do specific technical processing on the traffic data to ensure the validity of the adversarial sample.
2. We introduce the genetic algorithm into the method of generating the adversarial sample and realize the black-box attack against the machine learning model.
3. Our approach is equally valid for networks that have difficulty computing gradients or expressing mathematically.

The rest of the paper is organized as follows. Section 2 introduces the related work of adversarial samples. Section 3 presents TLTD (testing framework for learning-based IoT traffic detection system). Section 4 presents and discusses our experimental results. Finally, further discussions and conclusions are accomplished in Section 5.

## 2. Related Work

Given a primitive input (*X*), a target tag (*t*) and L(x)!=t, a similar input $X^{\prime }$ is found to make $L(X^{\prime })=t$. The special sample ($x^{\prime }$) with this characteristic is called an adversarial sample of the target attack. Similarity can be measured according to a distance algorithm [21]. Here are some common generation methods of adversarial samples and their application.

### 2.1. Fast Gradient Sign

Ian J. Goodfellow proposed a fast gradient sign to generate adversarial samples in 2015 [9]. The idea is to move every dimension of the sample to a small step toward decreasing confidence. Since input samples are usually multi-dimensional and activation functions ReLU (Rectified Linear Unit) are highly linear, such changes can affect the classification results.

The disturbance function is as follows:

(1) $$\eta =\epsilon \;sign({▽}_{x}J\left(\theta ,x,y\right))$$

where θ is the parameter of the model, *x* is the input of the model, *y* is the output class relative to *x*, and J(θ,x,y) is the loss function in the neural network.

So, the perturbed adversarial samples are

(2) $$x+\epsilon \;sign({▽}_{x}J\left(\theta ,x,y\right)),$$

where ϵ is set to be small enough to be difficult to distinguish. From an intuitive point of view, the fast gradient sign uses the gradient of the loss function to determine which direction each pixel should ultimately change in order to minimize the loss function. In the end, all pixels change in a certain direction by a certain size.

### 2.2. One Pixel Attack

Su et al. proposed one-pixel attack, which is an adversarial sample generation method based on differential evolution [22]. The typical adversarial sample generation method allows perturbation of all pixels; however, the approach considered by this method is reversed, focusing only on the number of modified pixels without limiting the size of a single variation.

(3) $$min\;loss_{F,t}\left(x^{\prime }\right),x^{\prime }\in {\left[0,1\right]}^{n}$$

(4) $$subject\;to\;\;∥x-x^{\prime }{∥}_{0}\leq d$$

where *d* is an integer—d=1 in the case of a single pixel attack.

Evolutionary algorithms do not require the gradient of the model to be solved. This is an advantage of this type of algorithm. However, this method is actually focused on solving the problem of single-pixel adversarial sample generation. The visual effect of the sample is not optimized. Adversarial samples have significant noise compared to the original sample.

### 2.3. Application of Adversarial Samples in Malware Detection

The way to generate adversarial samples by adding disturbance cannot only be applied in the fields of computer vision, speech, and natural language, but also in the field of network security. For example, when the adversarial sample is applied to the malware classification model based on machine learning, a slight disturbance is added to the malicious software without changing the attackability of the malware so that the classification model in the machine learning is misjudged to be the normal software.

Kathrin Grosse and Nicolas Papernot et al. applied the method of generating adversarial samples in the field of computer vision to malware classification [14]. In their method, the feature of the input sample is represented by a binary vector. Given a number of behavioral features (1,…,M), a software application can be represented by a set of binary vectors ($X\in {\left\{0,1\right\}}^{M}$), where $X_{i}$ indicates whether the *i*th behavior is allowed. For a single input sample *X*, the classifier returns a two-dimensional vector $F\left(X\right)=[F_{0}\left(X\right),F_{1}\left(X\right)]$, where $F_{0}\left(X\right)$ indicates that the software is normal software probability, and $F_{1}\left(X\right)$ indicates that the software is a malware probability and satisfies the constraint $F_{0}\left(X\right)+F_{1}\left(X\right)=\;1$.

The gradient of the input sample is calculated by the classification result to find the direction that most likely causes the classification result to change, as follows:

(5) $$J_{F}=\frac{\partial F(X)}{\partial X}={\left[\frac{\partial F_{i}\left(X\right)}{\partial X_{j}}\right]}_{i\in 0,1,j\in [1,m]}.$$

The method in this paper enables the classifier to misjudge malware as normal software, and its success rate is about 85%.

Compared to image data, there are the following differences in adversarial samples against malicious traffic or software:

(1) The input samples in the image are all pixels and the values of the pixels are continuous. However, in the field of network security, input characteristics are usually discrete and the range of values of different features is usually different.
(2) The pixels in the image can be freely changed within the value range. The restrictions on the modification of traffic or software are much more demanding. Arbitrary modifications may result in traffic or software not working properly.

## 3. Methodology

### 3.1. Framework

**Figure 1:**
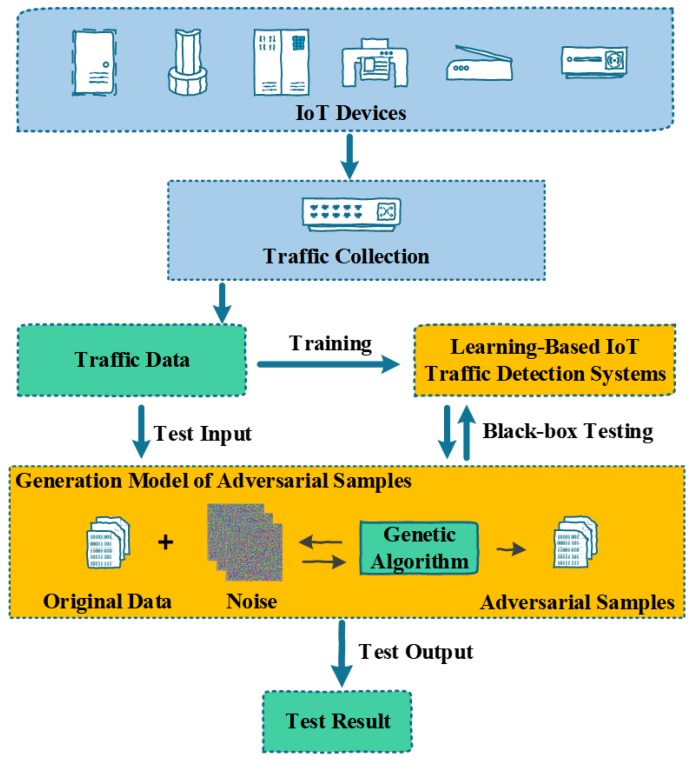
Overview of our testing framework for learning-based IoT traffic detection systems.

TLTD can detect the captured IoT traffic. The overview of TLTD is shown in Figure 1. The captured flow data and detection results are entered into our testing framework as test samples. Through multiple iterations, TLTD generates adversarial samples for the system, and then the system is tested for security. It can be seen that our testing framework can be well integrated with machine learning-based IoT traffic detection systems. In fact, our testing framework is suitable for the testing of a wider range of machine learning-based network traffic detection model systems, but we only use IoT traffic detection as an example. When the test results show that the detection system cannot resist the attack against the adversarial sample, this indicates that the system has potential safety hazards and it is necessary to implement such reinforcement measures as a distillation defense on the detection system. As we can see, determining how to generate an adversarial sample against IoT traffic is the main challenge of this testing framework. Therefore, we describe, in detail, the algorithm for generating an adversarial sample for IoT traffic.

### 3.2. Algorithm

The goal of the adversarial sample generation algorithm is to add a slight disturbance to the captured malicious IoT traffic so that the previously trained detection model misjudges it as normal traffic. Similar to the aforementioned adversarial sample for the malware detection model, for one input sample (*X*), the classifier returns a two-dimensional vector $F\left(X\right)=[F_{0}\left(X\right),F_{1}\left(X\right)]$, where $F_{0}\left(X\right)$ indicates the probability that the traffic is normal traffic, and $F_{1}\left(X\right)$ indicates the probability that the traffic is malicious traffic and satisfies the constraint $F_{0}\left(X\right)+F_{1}\left(X\right)=1$. The aim is to add a perturbation (δ), so that the classification result $F_{0}\left(X+\delta \right)$ of malicious samples (*X*) is less than $F_{1}\left(X+\delta \right)$, and the smaller the perturbation δ is, the better. This is equivalent to minimizing the number of disturbances and minimizing the degree of perturbation of each feature. In order to ensure the effectiveness of the traffic after the disturbance, we limited the type and amplitude of disturbance features. Details are described in Section 4. The difference is that we used genetic algorithms to select the degree of perturbation of the feature and thus realized black-box attacks against the machine learning model. The pseudo code of our algorithm is shown in Algorithm 1.

**Algorithm 1** Generating an adversarial sample.**Require:** Population Size pop_size, Number of features feat_num, Original sample $X_{i}$    $P_{i}\leftarrow initialization\left(\right)$    **for**
i=0→pop_size
**do**       $P_{i}\leftarrow Crossover\_Operator\left(\right)$       $P_{i}\leftarrow Mutation\_Operator\left(\right)$       **for**
j=0→feat_num
**do**          **if**
$P_{i}^{j}>0$
**then**             **Compute**
$\;\delta_{i}=P_{i}^{j}\left(upper_{i}^{j}-X_{i}^{j}\right)$         **else**             **Compute**
$\;\delta_{i}=P_{i}^{j}\left(X_{i}^{j}-lower_{j}\right)$         **end if**       **end for**       **Compute**
$\;\to X_{i+1}^{j}=X_{i}^{j}+\delta_{i}$       **if**
$F\left(X_{i+1}\right)<1-F\left(X_{i+1}\right)$
**then**          **Continue**       **else**          **Output**
$\;\to P_{i}$       **end if**    **end for**

For example, the original sample is

(6) $$X_{i}=\left[X_{i}^{0},X_{i}^{1},\ldots ,X_{i}^{n}\right].$$

One of the individuals in the genetic algorithm is

(7) $$P_{i}=\left[P_{i}^{0},P_{i}^{1},\ldots ,P_{i}^{n}\right].$$

The range of values for each dimension in the original data is

(8) $$R_{j}=\left[lower_{j},upper_{j}\right],j\in \left[0,n\right].$$

The range of values for each dimension in $P_{i}^{j}$ is [−1,1] are now specified. When $P_{i}^{j}$ is 0, it means that the i-th feature of the original sample does not mutate. When $P_{i}^{j}$ is negative, the original sample changes toward the smaller direction, and the degree of change is δ:

(9) $$\delta_{i}=P_{i}^{j}\left(X_{i}^{j}-lower_{j}\right),j\in \left[0,n\right].$$

When $P_{i}^{j}$ is positive, the original sample changes in the direction of increasing, and its degree of change is δ:

(10) $$\delta_{i}=P_{i}^{j}\left(upper_{i}^{j}-X_{i}^{j}\right),j\in \left[0,n\right].$$

So, the formula for the perturbed data is

(11) $$X_{i}^{j}=\left\{\begin{array}{cc} X_{i}^{j}+P_{i}^{j}\left(X_{i}^{j}-lower_{j}\right) & P_{i}^{j}>0\\ X_{i}^{j}+P_{i}^{j}\left(upper_{i}^{j}-X_{i}^{j}\right) & P_{i}^{j}\leq 0. \end{array}\right.$$

Finally, we get the formula for the fitness function in the genetic algorithm:

(12) $$F\left(P_{i}\right)=\omega_{d}D\left(P_{i}\right)+\omega_{e}\left(1-E\left(P_{i}\right)\right),$$

where $\omega_{d}$ and $\omega_{e}$ are two parameters. $D(P_{i})$ is the degree of difference between the original sample and the adversarial sample. $E(P_{i})$ is the success rate of adversarial samples in learning-based models.

**Figure 2:**
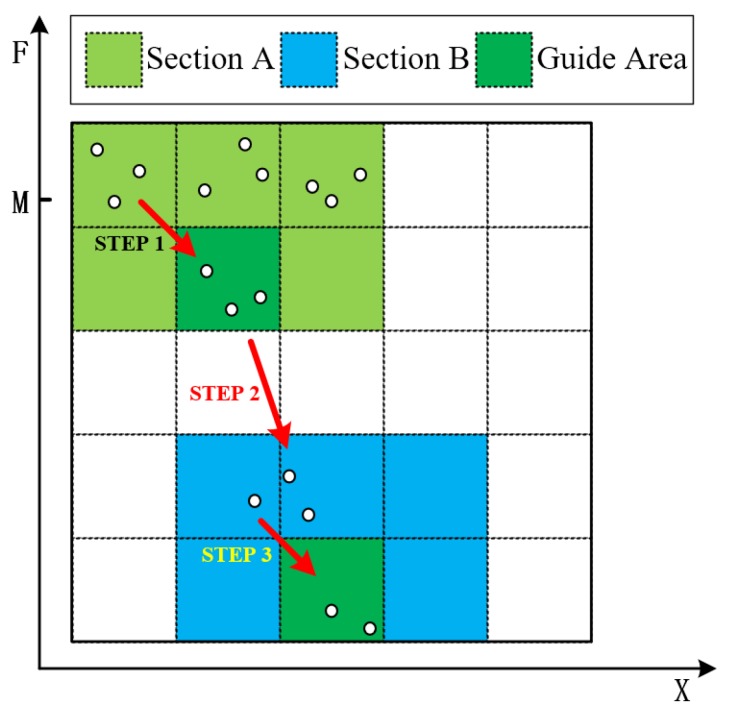
Individuals’ Distribution Diagram.

Through the preceding fitness function, the population (*P*) is actually divided into two sections, as shown in Figure 2. The whole optimization process can be divided into three steps.

Step 1. At this time, the adversarial sample cannot successfully mislead the classifier. Individuals at the top of section A gradually approach the bottom through crossover and mutation operators.
Step 2. The individuals move from Section A to Section B, indicating that $E(P_{i})=1$, i.e., the adversarial samples generated at this time can successfully mislead the classifier.
Step 3. Individuals at the top of Section B gradually approach the bottom, indicating the improvement of the similarity between the adversarial traffic and the original traffic.

Eventually, the bottom individual of Section B becomes the optimal individual in the population, and the information that it carries is the adversarial sample being sought out.

## 4. Experiments

### 4.1. Data Set and Environment

The dataset used in our experiments was KDD99 (Knowledge Discovery and Data Mining). Each sample in KDD99 contains 41 features. The 41 features can be divided into three categories:

1. Basic features of individual TCP connections;
2. Content features within a connection suggested by domain knowledge;
3. Traffic features computed using a two-second time window.

The hardware environment and software environment for all experiments are shown in Table 1.

### 4.2. IoT Traffic Detection Model

First, the detection model is trained to determine whether the traffic generated by the IoT device is malicious traffic. When the detection model reaches a certain accuracy, it combines with the method proposed in this paper to generate an adversarial sample and test the security of the model. Because the amount of data between different categories in KDD99 has changed significantly, we selected 4 categories with a large amount of data for perturbation, as shown in Table 2.

Neural network models are established for each of satan, ipsweep, portsweep, and nmap to find the recognition results. The detection model uses a fully connected network with a network structure of 32×64×2. The last layer output 0 indicates that the data is identified as a normal traffic type, and output 1 indicates the specified traffic type.

Finally, the detection rate of the detection model is as shown in the Table 3.

### 4.3. Simulation Experiments

We conducted a total of three sets of simulation experiments. There were slight differences in the technical implementation methods for generating adversarial samples in the simulation experiments. In each set of simulation experiments, we tested the four types of traffic detection models respectively. We hoped to select the best technical implementation by comparing the test results.

The experimental parameters are shown in Table 4.

The tournament selection strategy takes a certain number of individuals from the population each time, and then selects the best one of them into the offspring population. This operation is repeated until the new population size reaches the original population size. The specific steps are as follows:

STEP 1. Determine the number of individuals selected each time;
STEP 2. Choose individuals randomly from the population and select the individuals with the best fitness values to enter the offspring population;
STEP 3. Repeat STEP 2 for several times and the resulting individuals constitute a new generation of the population.

#### 4.3.1. TLTD-I

(A) Preprocessing

The numerical distribution of 13 features in the data set is more concentrated and less variable. If we disturb these features, we will destroy the inherent distribution of traffic characteristics. This makes the generated adversarial sample clearly distinguishable from normal traffic. In order to ensure the effectiveness of the countermeasures, we chose to eliminate these 13 data features; that is, the values of these 13 features were not changed when disturbed. The 13 features which were not used in TLTD-I are as follows: ***hot, num_failed_logins, logged_in, num_compromised, root_shell, su_attempted, num_root, num_file_creations, num_shells, num_access_files, num_outbound_cmds, is_hot_login, is_guest_login***.

(B) Fitness Function

The fitness function in TLTD-I is shown in Equation(12). We used the Euclidean distance to describe the degree of difference between the original sample and the adversarial sample:

(13) $$D\left(P_{i}\right)=\sqrt{sum_{j}^{n}{\left(X_{i}^{j}-X_{i}^{j^{\prime }}\right)}^{2}},j\in \left[0,n\right].$$

(C) Experimental Results

**Figure 3:**
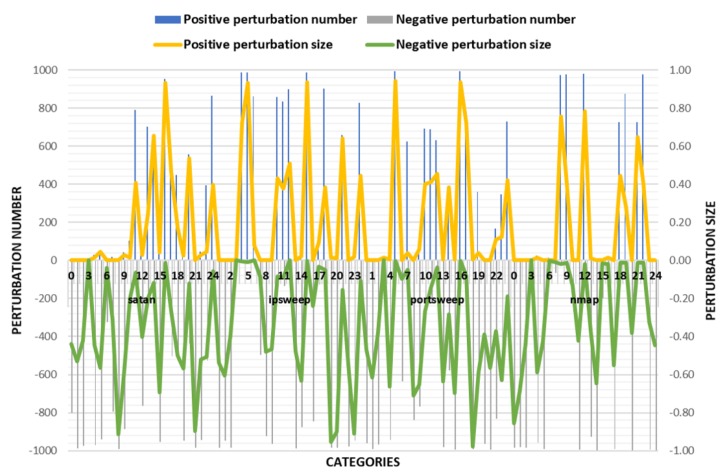
Comparison of the number of perturbation features and the size of the disturbance in TLTD-I.

The experimental results are shown in the Figure 3 and Table 5. According to the success rate of adversarial samples, the success rate of the other three classes was over 95% except for the low success rate of the adversarial samples on nmap traffic. However, there were very large changes in the values of some features in the disturbed flow data. Combining with actual scenarios, when these values change drastically, it is very likely to undermine the effectiveness of IoT traffic. Therefore, TLTD-I may not be suitable for practical applications.

#### 4.3.2. TLTD-II

(A) Data Preprocessing

In TLTD-II, a zero-mean normalization method was used to normalize all original data sets to a data set with a mean of 0 and a variance of 1.

The standardized formula is

(14) $$z=\frac{x-\mu }{\sigma },$$

where *z* is the normalized value, μ is the mean of the original data, and σ is the standard deviation of the original data. In addition, in order to simulate a more realistic IoT traffic detection environment, we also removed some of the data features that are difficult to obtain in real-world scenarios. Finally, we perturbed 22 features of the data.

(B) Fitness Function

In TLTD-II, we improved Equation (12) as the following equation:

(15) $$F\left(P_{i}\right)=e^{\frac{D(P_{i})}{\omega_{d}}}+e^{\frac{E(P_{i})}{\omega_{e}}}.$$

When the original sample is quite different from the disturbed sample, the left part has a larger value. In order to reduce the value of $F(P_{i})$, the left part is the leading factor in the optimization of the whole objective function. When the classification results of the neural network model do not change, the value of the right half will be very large. In order to reduce the value of the fitness function, the right half becomes the leading factor. In this case, the fitness function will be optimized in the direction of less difference from the original sample, and at the same time, the recognition result of the classifier will be changed. In brief, this improved fitness function satisfies the requirements of evolution orientation mentioned in the Section 3.2. On the other hand, its gradient is dynamic in the process of optimization, and it can find the optimal solution faster.

(C) Experimental Results

**Figure 4:**
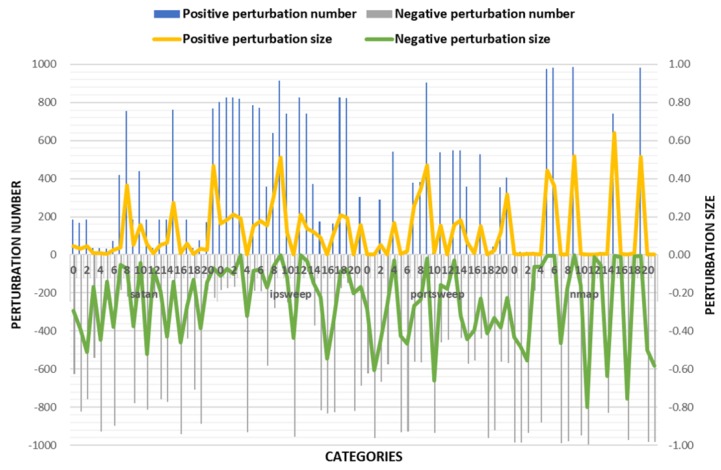
Comparison of the number of perturbation features and the size of the disturbance in TLTD-II.

The experimental results of TLTD-II are shown in Figure 4 and Table 6. Compared with TLTD-I, the average $D(P_{i})$ and the number of modified features were significantly reduced in TLTD-II. This means that we generated adversarial samples with fewer perturbations and fewer feature values. However, it also led to a bad result, that is, the success rate of the adversarial sample became very low. So, we propose TLTD-III and hope that the success rate can be further increased on the basis of TLTD-II.

#### 4.3.3. TLTD-III

**Figure 5:**
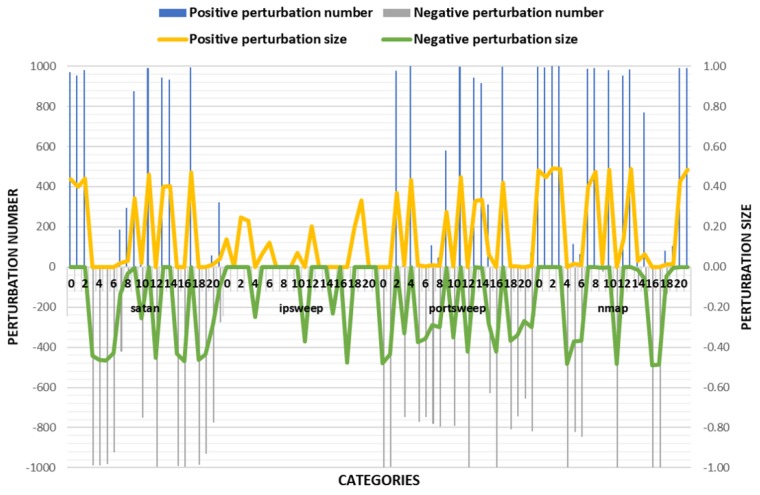
Comparison of the number of perturbation features and the size of the disturbance in TLTD-III.

In TLTD-III, the data pre-processing process and the fitness function were all those conceived in TLTD-II. We believe that the very low success rate of TLTD-II may be related to the large range of feature disturbances. In addition, in order to make the adversarial samples more similar to the original data, we defined the maximum range of variation for each type of feature as [−50%, 50%]. The experimental results are shown in Figure 5 and Table 7.

From the experimental results, it can be seen that the success rate of TLTD-III was almost 100% of the average of $D(P_{i})$ and the number of modified features was also small. This shows that TLTD-III has a certain application value in the actual scene.

### 4.4. Discussion

**Figure 6:**
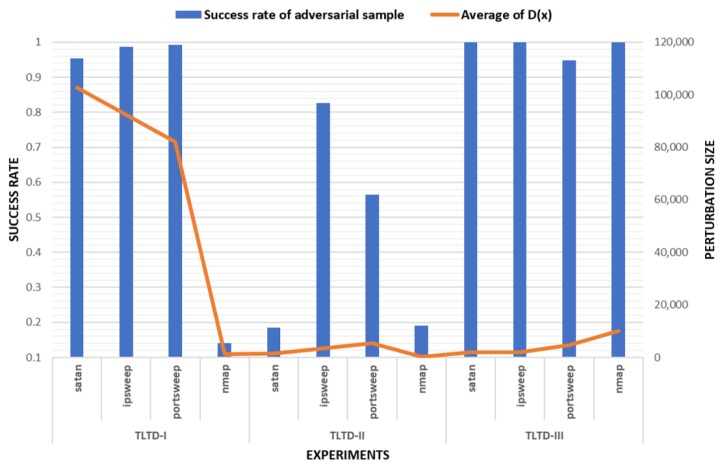
Comparison of the results of three experiments.

We compared the average success rate of adversarial samples and the perturbation size in three experiments. The results re shown in Figure 6. Compared with TLTD-I and TLTD-II, by modifying the normalization method, removing the unrelated feature data, and limiting the range of disturbance, we realized the dual purpose of a high success rate and low disturbance in TLTD-III. Although TLTD-III is still unable to ensure that the generated adversarial samples maintain the functionality of the original samples, we can still say that TLTD-III is an effective testing framework for learning-based IoT traffic detection systems.

## 5. Conclusions

To address the challenge of a lack of the testing framework for learning-based IoT traffic detection systems, we developed TLTD. Our experimental results show that our approach generates high-quality adversarial samples with the success rate closed to 100%. In the technical implementation of the TLTD algorithm, the selection of normalization method, features, and the range of disturbance are the keys to a good result. We hope TLTD can be a benchmark learning-based IoT traffic detection model. Our future work includes designing an effective defense approach to reinforce the traffic detection model.

## Figures and Tables

**Table 1:** The environment of all experiments.

**CPU (Central Processing Unit)**	Inter(R) Core(TM) i507400 CPU @ 3.00 GHz
**Memory**	8 GB
**Video Card**	Inter(R) HD Graphics 630
**Operating System**	Windows 10
**Programming Language**	Python 3.6
**Development Platform**	Jupyter Notebook
**Dependence**	Tensorflow, numpy etc.

**Table 2:** The categories we selected in KDD99 (Knowledge Discovery and Data Mining).

Category	Satan	Ipsweep	Portsweep	Nmap
**Amount**	15,892	12,381	10,413	2316

**Table 3:** The detection rates of the models.

Category	Satan	Ipsweep	Portsweep	Nmap
**Detection Rate**	0.9940	0.9805	0.9931	0.9330

**Table 4:** The parameters of TLTD.

Population	Cross Probability	Mutation Probability	Selection	Iterations	$\omega_{d}$	$\omega_{e}$
300	0.5	0.3	Tournament	200	1000	150

**Table 5:** The results of TLTD-I. The data in the table is the average of the 1000 sample test results.

Category	Success Rate	Average of $P_{i}$	Average of $D\;(P_{i})$	The Number of Modified Features
**satan**	0.953	−0.139	102,729.98	21.493
**ipsweep**	0.986	0.352	92,384.84	21.975
**portsweep**	0.993	−0.117	82,101.05	22.459
**nmap**	0.140	−0.072	1337.47	18.918

**Table 6:** The results of TLTD-II. The data in the table is the average of the 1000 sample test results.

Category	Success Rate	Average of $P_{i}$	Average of $D\;(P_{i})$	The Number of Modified Features
**satan**	0.185	−0.177	1479.37	17.441
**ipsweep**	0.826	0.309	3322.13	20.431
**portsweep**	0.564	−0.197	5341.48	18.841
**nmap**	0.190	−0.148	186.95	16.807

**Table 7:** The results of TLTD-III. The data in the table is the average of the 1000 sample test results.

Category	Success Rate	Average of $P_{i}$	Average of $D\;(P_{i})$	The Number of Modified Features
**satan**	1	−0.062	1888.23	19.765
**ipsweep**	1	0.379	1868.63	19.943
**portsweep**	0.949	−0.118	4622.63	19.554
**nmap**	1	0.098	3010.94	18.276
